# Successful Treatment of Refractory Palmoplantar Pustular Psoriasis With Apremilast: A Case Series

**DOI:** 10.3389/fmed.2020.543944

**Published:** 2020-10-15

**Authors:** Sascha Ständer, Felicia Syring, Ralf J. Ludwig, Diamant Thaçi

**Affiliations:** ^1^Department of Dermatology, University of Lübeck, Lübeck, Germany; ^2^Lübeck Institute of Experimental Dermatology, University of Lübeck, Lübeck, Germany; ^3^Institute and Comprehensive Center for Inflammation Medicine, University of Lübeck, Lübeck, Germany

**Keywords:** psoriasis, apremilast, pustular palmoplantar psoriasis, treatment, case series

## Abstract

**Introduction:** Palmoplantar pustular psoriasis (PPPP) is a debilitating inflammatory skin disorder of the palms and soles that poses a high burden on affected patients. Satisfactory treatment response is rarely achieved using current treatment options, little is known about the potential benefit of the PDE4 inhibitor apremilast in the treatment of refractory PPPP patients. We aimed to evaluate the use of apremilast in PPPP patients.

**Patients and Methods:** Six patients, four with severe physician global assessment (PGA) = 3 on a scale of 0–4 and two with very severe (PGA = 4) treatment-refractory PPPP [mean age (years ± SD): 56.2 ± 15.6], were included in this study. Five patients had concomitant psoriatic arthritis (PsA). Prior to apremilast administration, topical corticosteroids, psoralen-UVA and multiple systemic oral and biologic anti-inflammatory treatments were insufficient to improve their skin condition or had to be discontinued due to adverse events. Apremilast (titrated to a maintenance dose of 30 mg 2x/d) was commenced in all patients with clinical follow-up over 18 months.

**Results:** Within the first 4 weeks of treatment, each patient's symptoms improved as assessed by PGA score. At 3 months, four patients had a mild PGA score and two were cleared from PPPP. After 18 months of follow-up, three patients improved from PGA = 3 to PGA = 1 and one patient from PGA = 4 to PGA = 1. Two patients discontinued treatment, one due to a lack of efficacy against PsA and the other to a desire to have a child. However, both patients recorded improvements before discontinuing treatment.

**Conclusion:** Apremilast may be a promising treatment option for refractory and severely affected PPPP patients. Our observation, however, requires further validation.

## Introduction

Palmoplantar pustular psoriasis (PPPP) is a chronic inflammatory IL-17/23-pathway- driven skin condition characterized by the development of sterile pustules on the palms and soles (1). The prevalence ranges from 0.01 to 0.05% in Western Europe and North America with slightly higher rates of ~0.12% in Japan. PPPP predominantly affects females who smoke and is associated with a relatively high rate of concomitant arthritis (2, 3). PPPP leads to debilitating skin conditions and significantly impaired quality of life. Additionally, PPPP patients report higher usage of potent topical anti-inflammatory treatment compared with patients with moderate-to-severe plaque-type psoriasis (4).

Despite the debilitation effects of this disease, insights into the pathogenesis of PPPP are scant. Several studies indicate a dominant role of IL-17 and IL-22 in palmoplantar pustulosis (5, 6). Pathogenetic hallmarks of PPPP include increased cutaneous expression of IL-17A and, in contrast to psoriasis, lower IL-23 expression (7). Since the driving pathologic mechanisms of the disease are not well illuminated, treatment of PPPP is often challenging. Although medications used for the management of psoriasis vulgaris generally do not have regulatory approval for PPPP, they are often used in these patients due to limited treatment options. Current treatments for PPPP, based on low evidence levels and expert opinions, [e.g., phototherapy, cyclosporine A (CsA) and topical corticosteroids], result in remission in some patients (8). However, prolonged immunosuppression is required to maintain remission and relapses occur frequently. Additionally, treatment-related adverse events and morbidity further add to the patients' burden and to a reduced quality of life. Hence, investigation of novel effective and safe treatment options is highly warranted.

Apremilast is a small molecule oral phosphodiesterase (PDE) 4 inhibitor that has been approved for moderate-to-severe plaque psoriasis and psoriatic arthritis (9). On a molecular level, apremilast prevents cAMP hydrolysis which leads to increased intracellular cAMP levels and down-stream signaling with subsequent reduction of NF-kappa-B-dependent anti-inflammatory signaling (e.g., protein kinase A mediated release of IL-10) (10, 11). Randomized trials and real-world studies have recently documented the efficacy and safety of apremilast in psoriasis patients (12–15). While safety has been proven in numerous patients, the efficacy of apremilast in PPPP has not been well-studied and data on the use of apremilast in both treatment-refractory and treatment-naïve PPPP patients are scarce. Hence, to add further insight into the use of apremilast in PPPP patients, we retrospectively analyzed the effectiveness of apremilast administration in severe to very severe PPPP patients refractory to multiple prior topical and systemic treatments.

## Patients and Methods

Six patients [mean age (years ± SD): 56.2 ± 15.6, four female, two male] with refractory PPPP [mean disease duration (years ± SD): 13.7 ± 10.6] were included. Four patients were observed for 18 months and two for 6 months. Data were analyzed retrospectively. Five patients had concurrent psoriatic arthritis (PsA). Diagnosis of PsA was based on the fulfillment of the CASPAR classification criteria and confirmed by our in-house rheumatologists. The patients' characteristics are summarized in Table 1. All six patients had been previously treated with locally applied highly potent glucocorticosteroids (GCs), partially under occlusion, psoralen-ultraviolet A (UVA), methotrexate (MTX) and at least four different systemic anti-inflammatory drugs prior to initiation of apremilast treatment. Most of the patients were treated with oral retinoids [acitretin (5/6) or alitretinoin (2/6)] (Table 2). Four patients received CsA and three were treated with biologics. In all individuals, the prior treatment had failed or was discontinued due to adverse effects. Apremilast treatment was commenced with an initially low and subsequently increasing dose until the maintenance dose of 30 mg 2x/d was reached. Clinical condition was scored by physician global assessment (PGA) as PGA = 0 [cleared (0 pustules)], PGA = 1 [mild (1–3 pustules)], PGA = 2 [moderate (3–10 pustules)], PGA = 3 [severe (10–20 pustules)], PGA = 4 [very severe (>20 pustules)] within a region effected by pustules e.g., palms or/and soles. Ethical approval was not required f or the retrospective analysis of these.

**Table 1:** Characteristics of six treatment refractory PPPP patients treated with apremilast.

**Case**	**Age (years)**	**Sex**	**PGA at baseline**	**PGA at follow-up endpoint**	**Follow-up (months)**	**Disease duration (years)**	**Smoking history**	**Concomitant disease**
1	76	Female	3	1	18	33	positive	PsA, fibromyalgia
2	56	Female	4	1	18	7	negative	PsA, arterial hypertension, type II diabetes, depression, obesity
3	48	Male	3	1	18	6	positive	Depression, arterial hypertension
4	36	Female	3	0	6	12	positive	PsA
5	73	Female	3	1	18	18	positive	PsA, arterial hypertension, Hypothyreosis, Asthma
6	48	Male	4	0 (soles) 1 (palms)	6	6	positive	PsA, depression

**Table 2:** Prior treatment before the treatment with apremilast.

**Case**	**Acit**	**Alit**	**PUVA**	**MTX**	**CsA**	**GCS**	**AZA**	**ETN**	**UST**	**ADA**	**SEC**
1	x		x	x	x						
2	x		x	x	x	x		x	x	x	
3	x	x	x	x							
4			x	x	x	x	x				x
5	x	x	x	x		x					
6	x		x	x	x	x					x

*Acit, acitretin; Alit, alitretinoin; PUVA, psoralen-UVA; MTX, methotrexate; CsA, cyclosporine A; GCs, glucocorticosteroids; AZA, azathioprin UST, ustekinumab; ADA, adalimumab; SEC, secukinumab*.

## Results

**Figure 1:**
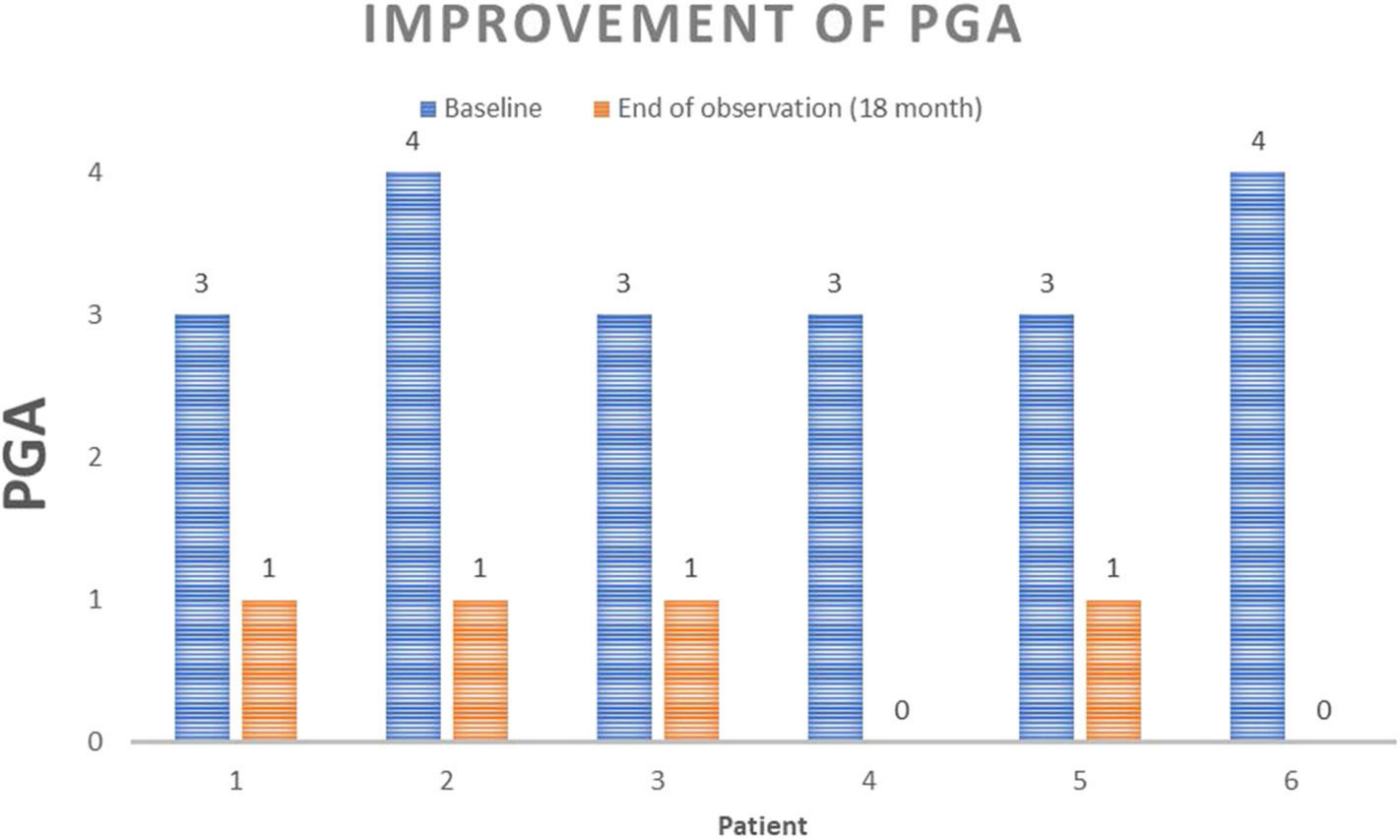
Improvement of pustular PGA at the end of observation time. Paired *t*-test *p* < 0.001.

After treatment initiation, the patients' skin condition improved within the first 4 weeks of treatment, from PGA = 4 in two cases and PGA = 3 in four cases to PGA = 1 in five cases and PGA = 2 in one case. After the 12-week follow- up visit, four patients had a PGA = 1 score and two had a PGA = 0 score. Four patients had a PGA = 1 score after 18 months, whereas two patients discontinued the drug after 6 months, one due to a lack of effect on joint involvement and the other due to the desire for children (Figure 1). In all six patients, adverse effects were mild and included tolerable gastrointestinal side effects (e.g., nausea, diarrhea) after the first oral apremilast doses. No severe adverse events under apremilast were observed.

**Figure 2:**
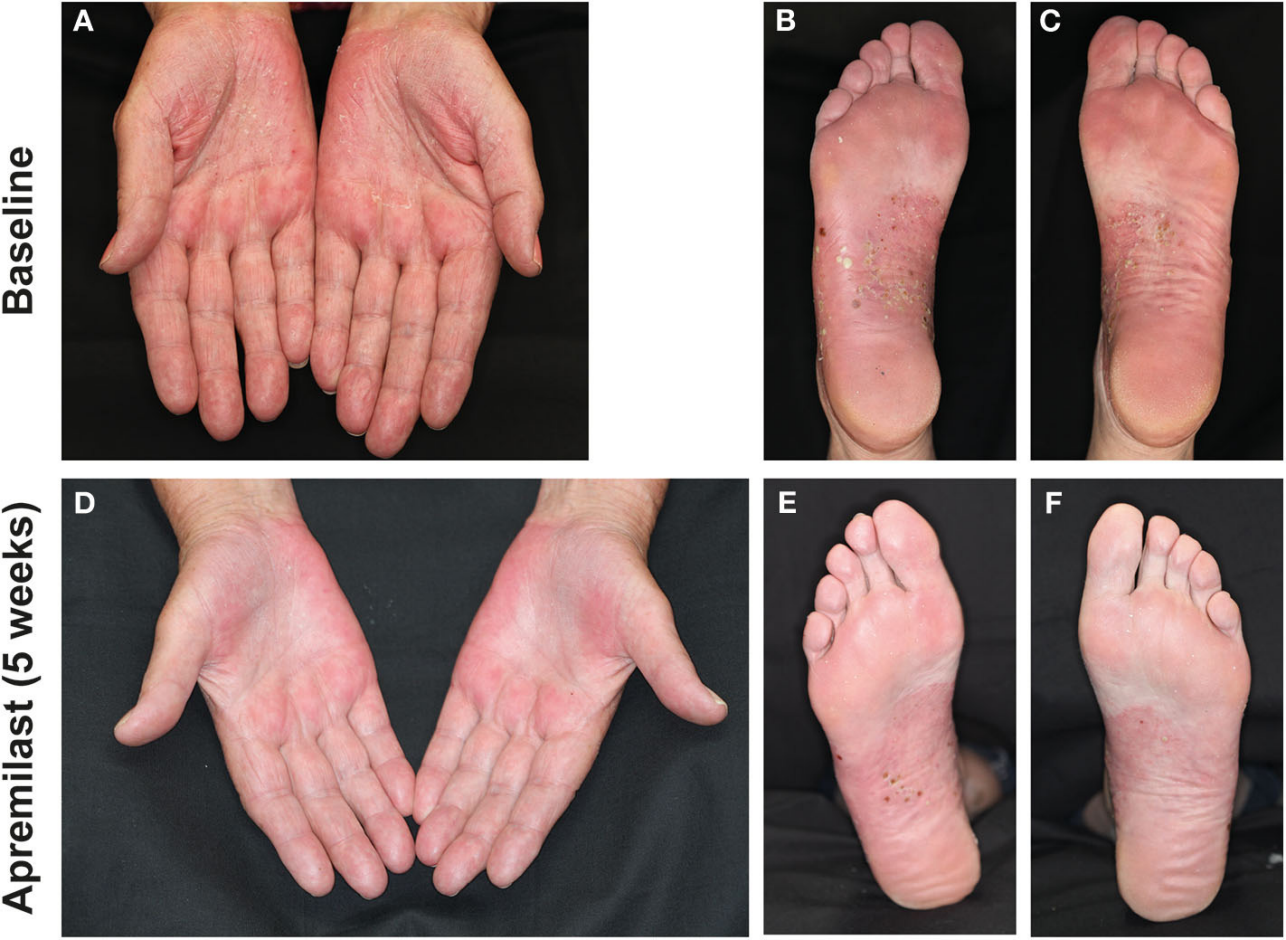
Palms and soles of patient 1 with numerous pustules on scaled red skin at baseline **(A–C)** and after 5 weeks of apremilast treatment **(D–F)** with partial remission and reduced inflammation.

In more detail, **Patient 1** (75-year-old female) was diagnosed PPPP 33 years earlier. Concomitant diseases were PsA and fibromyalgia. Previous treatment of PPPP included systemic acitretin (20 mg/day) in combination with psoralen-UVA, MTX (15 mg/week s.c.), and CsA (3 mg/kg/bw). These treatments were not tolerated and were therefore discontinued. Five weeks after apremilast initiation, the patient's PsA improved substantially and her PPPP was scored as PGA = 1 after an initial PGA = 3 (Figure 2). At the follow-up visit after 5 months, the patient presented with a relapse of PPPP following discontinuation of apremilast 3 weeks before the visit (relapse experienced 2 weeks after discontinuation and 1 week before the visit). Several days after apremilast was re-administered, she achieved a score of PGA = 1 that remained stable at subsequent visits.

**Figure 3:**
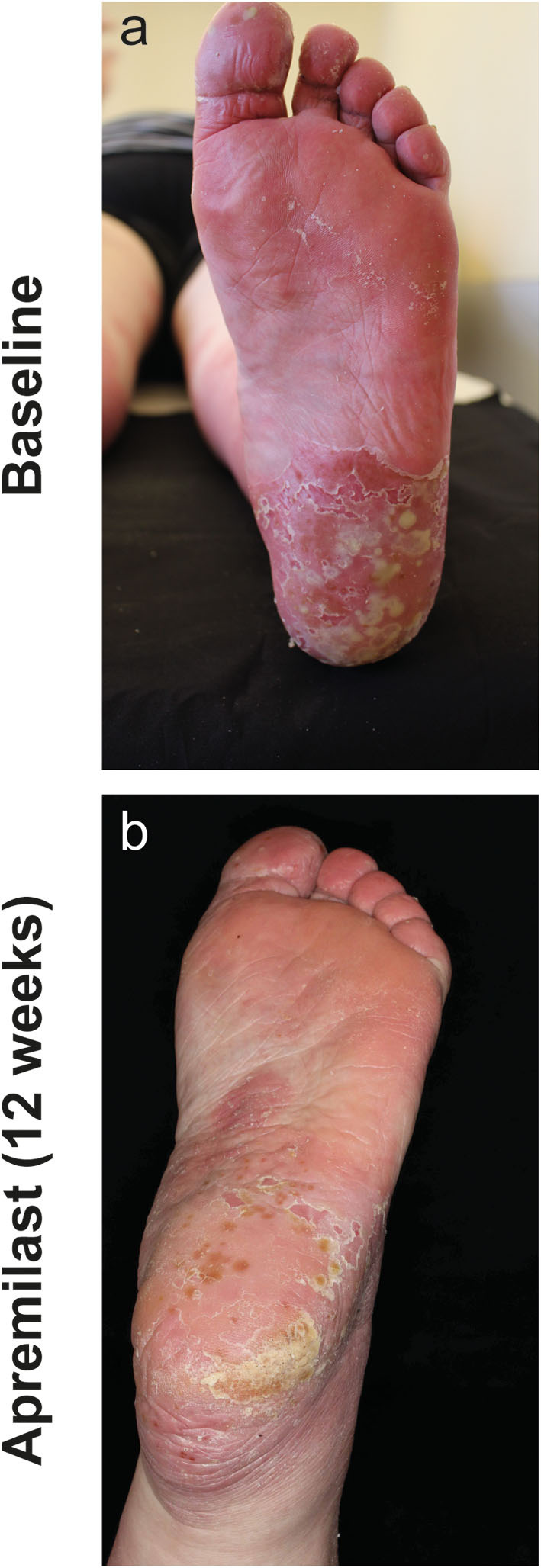
Left sole of patient 2 with numerous conflating pustules on inflamed skin at baseline **(a)** and after 12 weeks of apremilast treatment **(b)** with substantial decrease of inflammation and pustules.

**Patient 2** (55-year-old female) was diagnosed with PPPP 7 years before presentation. Concomitantly, she suffered from PsA, arterial hypertension, depression, obesity and type II diabetes. Previous treatment with acitretin (30 mg/d), psoralen-UVA, CsA (300 mg/day), adalimumab, ustekinumab, and secukinumab did not improve the skin lesions. Due to adverse effects (e.g., infections), biologic treatments were discontinued. After apremilast initiation, the patient reported rapid subjective relieve of symptoms within several days. After 4 weeks, pustules decreased in number and size and disease activity improved from a PGA = 4 to a PGA = 2. After 3 months of treatment with apremilast and at all subsequent follow-up visits, the patient recorded a PGA = 1 (Figure 3).

**Patient 3** (54-year-old male) was diagnosed with PPPP 6 years earlier. Previous treatment included alitretinoin (30 mg/d), acitretin (50 mg/d), MTX (20 mg/week), and psoralen-UVA. None of these therapies was effective in inducing remission. The patient presented clinically with severe PPPP (PGA = 3). After apremilast initiation, the patient reported a significant subjective improvement of the skin within 10 days. However, the drug was discontinued due to a depressive episode after 4 months (no suicidal ideation) with a subsequent new onset of pustules within 2 days. After interdisciplinary psychiatric consultation, therapy with apremilast was reintroduced leading to major improvement of skin lesions within 1 week. The patient maintained a PGA = 1 at all following visits.

**Patient 4** (35-year-old female) was diagnosed with PPPP 12 years previously and had concomitant PsA. Previous treatments included oral GCs, psoralen-UVA, secukinumab, CsA (250 mg/d), azathioprine (dose not recalled), and MTX (20 mg/week s.c.). The patient suffered from severe skin involvement PGA = 3. Skin lesions improved within 2 weeks after apremilast initiation to PGA = 1. At the week 12 follow-up visit pustules on the palms and soles were observed to resolve completely (PGA = 0). However, apremilast was subsequently discontinued due to the patient's desire to have a child.

**Patient 5** (72-year-old female) was diagnosed with PPPP 18 years previously; she also had concomitant PsA. Previous therapies included oral GCs, MTX (25 mg/week), psoralen-UVA, alitretinoin (30 mg/d) and acitretin (25 mg/d). After initial paradoxical worsening of severe palmoplantar skin lesions to PGA = 3, improvement to PGA = 2 was observed after 4 weeks. At the next visit the patient recorded a PGA = 1 for the palms and PGA = 1–2 for the soles. The patient's skin condition remained stable at PGA = 1 for 18 months during ongoing apremilast treatment.

**Patient 6** (47-year-old male) was diagnosed with PPPP 6 years earlier and had concomitant PsA and depression. Previous treatment with acitretin (40 mg/d), psoralen-UVA, MTX (20 mg/week), CsA (up to 400 mg/d), oral corticosteroids GCs (up to 10 mg/d), and secukinumab (300 mg/month) were discontinued due to lack of efficacy and/or adverse effects. Due to highly painful joint involvement under the current therapy regimen, including combined secukinumab and CsA, the patient received prednisolone (10 mg/d) several weeks both before and initially during the first days of apremilast treatment. Four weeks after apremilast initiation, the patient reported clinical improvements to a PGA = 1. At the week 12 follow-up, disease activity decreased to PGA = 2 with five pustules on the palms. The soles displayed no pustules but slight desquamation on erythematous skin (PGA = 0). However, due to an exacerbation of PsA, apremilast was discontinued after 6 months in order to initiate TNF-alpha inhibitor treatment. At the time of apremilast discontinuation, the patient had cleared soles and a PGA = 1 of the palms. In all six patients, adverse effects were mild and included tolerable gastrointestinal side effects (e.g., nausea, diarrhea) after the first oral apremilast administrations. No severe adverse events under apremilast were observed.

## Discussion

Therapeutic approaches for PPPP have limited effectiveness and many patients remain refractory to all available agents; novel treatment options for this condition are greatly needed. Apremilast, a small-molecule PDE4-inhibitor, is a novel anti-inflammatory drug that has proven its efficacy in plaque type psoriasis and PsA over the past years, but has not been well-studied refractory PPPP. However, case reports have highlighted apremilast as an alternative in refractory and severe PPP patients. Controlled observations concerning the use of apremilast in PPPP, however, remain scant.

By inhibition of PDE4, apremilast increases intracellular cyclic AMP, which is an important second messenger in immune cells influencing inflammatory cascade (16). In PPPP, T-cells produce numerous cytokines, including TNF-a, IL-17, and IL-22, which stimulate keratinocytes to proliferate (17). Inhibition of proinflammatory cytokines such as IL-23 by apremilast decreases recruitment of Th1 and Th17 to the skin. In patients with moderate-to-severe psoriasis, treatment with apremilast was associated with significant reductions in plasma levels of interleukin (IL)-17F, IL-17A, IL-22, and TNF-α. Furthermore, PDE4 blockade inhibits neutrophil chemotaxis through decreased production of leukotriene B4 and IL-8 and prevents the migration of neutrophils to the epidermis (16). IL-36RN (receptor antagonist) gene mutation might play an important role in pustular forms of psoriasis like GPP (generalized pustular psoriasis) and acrodermatitis continua of Hallopeau. In contrast PPP seems not to be related to IL36 RN mutation and have a different pathogenesis from GPP (18). Since apremilast modulates both pro- and anti-inflammatory mediators it could explain in a part clinical efficacy in PPPP.

Our case series adds to the body of evidence on the use of apremilast in PPPP. To our knowledge, this is the first cases series to describe the successful use of apremilast in six severe, treatment-refractory PPPP patients, five of whom had concomitant PsA, over a period of 18 months. All the patients improved during apremilast treatment and there were no reports of severe adverse events under treatment.

These observations seem to be a first promising hint toward the use of apremilast in severe and refractory PPPP. Our data support the initiation of larger, randomized, controlled studies of apremilast in both treatment-naïve and treatment-refractory PPPP patients.

## Data Availability Statement

All datasets generated for this study are included in the article/supplementary material.

## Ethics Statement

Ethical approval for this study was not required in accordance with local legislation and national guidelines, as the study describes routine care which does not require ethical approval. Written informed consent was obtained from all participants for the publication of any identifiable images or data in the article.

## Author Contributions

DT designed the study. DT and FS treated and documented the patients. SS extracted all data from the electronic documentation. SS, FS, RL, and DT wrote the manuscript and contributed to the revision, read and approved the submitted version. All authors contributed to the article and approved the submitted version.

## Conflict of Interest

RL has received honoraria and/or research grants from the following companies: Admirx, Almirall, Amryth, ArgenX, Biotest, Biogen, Euroimmun, Incyte, Immungenetics, Lilly, Novartis, UCB Pharma, Topadur, True North Therapeutics, and Tx Cell. DT has received honoraria or fees for serving on advisory boards, acting as a speaker, or as a consultant, from AbbVie, Amgen, Almirall, Beiersdorf, Bioskin, Biogen, Boehringer Ingelheim, Celgene, Galapagos, GlaxoSmithKline, Dignity-Science, Leo Pharma, Medac, Merck Sharp & Dohme, Morphosys, Lilly, Novartis, Janssen, Pfizer, Regeneron, Sanofi, Samsung, Sandoz, Hexal, Sun Pharmaceuticals, UCB; and has received grants from Celgene and Novartis. The remaining authors declare that the research was conducted in the absence of any commercial or financial relationships that could be construed as a potential conflict of interest.
